# The Use of Drugs in Insomnia

**Published:** 1905-10-28

**Authors:** 


### THE USE OF DRUGS IN INSOMNIA.

Dr. Hale White,[1] after recounting various non-medicinal aids to the promotion of sleep, and advising that these be adopted rather than drugs in cases of chronic insomnia, recognises that in the presence of disease or in sleeplessness due to temporary anxiety or excitement the use of drugs may be necessary. He advises that medicines should not be given as compressed tablets, as these may either escape solution altogether or may dissolve very slowly, with a resulting postponement of the desired action. Of sleep-producing drugs chloral hydrate is widely popular, as it is easily taken and rarely produces any disagreeable after effects. As it depresses the action of the heart and lowers the blood-pressure, it ought not to be given in cardiac disease or in unduly low blood-pressure. Chloralamide is free from these disadvantages, and is an excellent hypnotic in cases of heart disease; it must be given in solution. The best method of administration is to put the desired dose (say 25 grains) in an ounce of brandy, stirring until the drug is dissolved, then adding water to taste, and taking the mixture about an hour before bedtime. If given as a powder, solution and absorption are apt to be so slow, that the effects are postponed for many hours. Paraldehyde is particularly valuable when insomnia is associated with delirium or with any mental aberration. It is soluble in water to the extent of 1 in 10, so that 1½ drachms may be given in 2 ounces of water, and the taste covered by tincture and syrup of orange; if necessary, it may be given per rectum. Paraldehyde does not depress the heart; it quickly produces refreshing sleep, but its odour, which can be recognised in the breath for several days after administration, forbids its use except in the case of patients confined to the house. Sulphonal, trional, tetronal, and veronal are all useful hypnotics which neither depress the heart nor upset the digestion. As they are insoluble, they should be given in a finely-divided condition and enclosed in a cachet, the dose being taken about an hour before bedtime and washed down with a draught of hot water. These drugs should not be used in cases of renal disease. Hyoscine should never be regarded as an ordinary hypnotic, but it may be used in insomnia accompanying severe illness, especially if mental disorder is present. It is contra-indicated by a weak pulse, a low blood-pressure, or laboured respiration.

[1] Brit. Med. Jour., October 21, 1905.

